# Audiological practice and COVID-19: recommendations that audiological centers can use to maintain the safety and quality of service—expert opinion

**DOI:** 10.1007/s00405-021-06766-w

**Published:** 2021-03-27

**Authors:** Ranjith Rajeswaran, Dayse Tavora-Vieira, Griet Mertens, Margaret Dillon, Saranya Narayan, Mohan Kameswaran, Anja Kurz

**Affiliations:** 1 Madras ENT Research Foundation (MERF), Chennai, Tamil Nadu India; 2 MERF Institute of Speech and Hearing (P) Ltd, Chennai, Tamil Nadu India; 3 Department of Otolaryngology, Head and Neck Surgery, University of Western Australia School of Medicine, Perth, Australia; 4 Department of Otorhinolaryngology, Head and Neck Surgery, Antwerp University Hospital (UZA), Antwerp, Belgium; 5 Department of Otolaryngology/Head and Neck Surgery, University of North Carolina at Chapel Hill, Chapel Hill, USA; 6 Neuberg Laboratories, Chennai, India; 7 University Hospital Würzburg, Department of Oto-Rhino-Laryngology, Plastic, Aesthetic and Reconstructive Head and Neck Surgery, Comprehensive Hearing Center, Josef-Schneider-Str. 11, 97080 Würzburg, Germany

**Keywords:** COVID-19, Audiological services, Cochlear implant, Hearing aid, Triage, Remote care/telehealth

## Abstract

**Purpose:**

Audiology is an essential service for some patient groups and some interventions. This article sets forth experience-based recommendations for how audiological centers can continue to safely and effectively function during COVID-19.

**Methods:**

The recommendations are the result of panel discussion and are based on the clinical experience of the panelists/authors.

**Results:**

The recommendations cover which patient groups and which interventions should be treated when and whether this can be performed in the clinic or remotely; how to maintain the safety of workplace via optimizing patient flow within the clinic and the sanitation of rooms and equipment; and overcoming communication challenges that COVID-19 intensifies.

**Conclusion:**

For essential audiological services to continue under COVID-19, safety measures must be implemented and maintained, and treatment and communication strategies must be adapted to offset communication difficulties due to personal protective equipment (PPE) and social distancing and to bolster patient confidence. In short, it is vital that staff feel safe, that patients either feel the clinic is safe enough to visit or that remote treatment may be an option, and that clinics and patients have a broad agreement on the urgency of any needed service. We hope that these recommendations help clinics effectively accomplish these goals.

## Introduction

We believe that hearing health care is an essential service for at least some patient groups and some interventions. We also believe that by rigorously following safety guidelines, essential hearing health care can be safely delivered to patients. Thus, the aim of this article is to provide recommendations that audiological centers can use to maintain the safety and quality of services during the COVID-19 pandemic. And by maintaining safety, allowing patients to confidently access and benefit from the hearing health care they need. These recommendations have been designed to be flexible enough so that they can be applied to different regions in the world and different phases of the pandemic.

## Methods

These recommendations were derived from a (virtual) panel discussion in June 2020 and are based on the clinical experience of the panelists.

The panelists are audiologists or surgeons and are members of the HEARRING group, which is a worldwide collaborative of more than 30 comprehensive hearing centers dealing with all aspects of hearing restoration with implantable devices [1].

### Recommendations

The foremost priority of all the recommendations herein is maintaining the health and safety of the health care providers. If they are not healthy, the health care system cannot function.

We have used polymerase chain reaction (PCR) testing as the means of diagnosis. Guidelines on the diagnosis of COVID-19 differ from country to country and from time to time; therefore, what constitutes a COVID-19 case will depend on these local guidelines. The focus of this article, however, is not the diagnosis (or treatment) of COVID-19, but rather to provide recommendations to limit any infection risks during routine audiology practice on patients who are not consciously aware that they are infected with COVID-19.

### Procedures: In person or remote? Urgent or postponable?

Every patient is important. All procedures, however, are not equally urgent in all patients. We have divided procedures and patients into 3 categories: urgent (ASAP), priority (within 4 weeks), and elective (can be postponed for 12 weeks or longer). These are general recommendations, actual circumstances in the clinic and its environment must be considered.

These recommendations are guided by 2 core ideas. First, the priority is to provide patients with, at minimum, audibility in 1 ear. Thus, device failure in a unilateral CI user with bilateral deafness is of greater priority than one device failure in a bilateral CI user. Second, reducing the number of patient visits (i.e., people coming into the clinic) can make it easier to keep the people within the clinic safe, thus, increasing the use of remote services is preferable. Not all procedures, however, can be done effectively in a remote setting (Table 1 here).

**Table 1:** List of interventions according to their suggested urgency and if they necessitate an in-person visit

	In-person visit is required	Remote care is recommended
*Urgent*	CI troubleshooting/repair for issues that cannot be resolved remotely Newborn hearing screening Audiological evaluation and counseling on CI candidates with meningitis, head trauma, or other pathologies with risk of cochlear ossification Audiological evaluation and counseling on CI revision due to infection Audiological evaluation and counseling on device failure in unilateral CI users who have profound bilateral deafness Acute vestibular function testing Audiological evaluation in cases of sudden deafness	First CI reprogramming after activation (this can be done remotely *if* the clinic has regular experience in this)
*Priority*	ABR in children HA fitting (for children and elderly people) Troubleshooting with children and elder people who are at risk of cognitive decline and/or social isolation with a HA for issues that cannot be solved remotely CI activation (first-fitting) if not possible to do it remotely Speech rehabilitation sessions in children Audiological evaluation and counseling on CI in children candidates with prelingual deafness (esp. before 3 years old and with bilateral deafness) Audiological evaluation and counseling on CI in elderly candidates with risk of cognitive decline and social isolation First 2–4 CI programming sessions after activation	General troubleshooting Counseling Speech rehabilitation Groups sessions of tinnitus retraining therapy Auditory training with adults
*Elective*	*In-person visit is required* General and routine audiological testing ABR (in adults) Routine vestibular testing Fine adjustments of HAs Fine adjustments of CIs Auditory processing disorder assessment Audiological evaluation and counseling on CI in children and adults with no evident risk of social isolation/cognitive decline Audiological evaluation and counseling for second implant *In-person visit is recommended* CI programming after the first 2–4 post-activation sessions	CI programming after the first 2–4 post-activation sessions can be done remotely *if* the clinic has regular experience in this

*ABR *auditory brainstem response, *CI *cochlear implant, *HA *hearing aid

### Precautions and recommendations

#### All employees of the clinic

- Should receive regular polymerise chain reaction (PCR) testing.
- Face masks are mandatory for entry into the building (Personal protective equipment for activities within the clinic is covered in in Sect. “Personal protective equipment (PPE))”.

#### Optimizing patient flow

- Patients should register and pay online, if possible.
- No accompanying people are allowed for adult patients unless they have ambulatory issues or are infirm; 1 accompanying adult is allowed for each child.
- Patients are only allowed inside during appointment time
- All patients and visitors should be screened upon arrival using a COVID-19 checklist.
- Patients may only enter via 1 designated entrance, i.e., there should not be multiple entry points; similarly, patients may only exit via 1 designated exit, which is not the same door as the entrance.
- If patients (or their accompanying person) are not wearing a mask or if they are wearing a mask that is not triple layered, they should be provided with, at minimum, a fresh surgical mask. Regulations vary according to country, but visitors should be provided with the safest mask possible.
- To help ensure patients go where they intend to go, the clinic should have sign boards and floor signs.
- Floor signs should also be used to remind patients to keep appropriate social distancing (of at least 1 m [2]).
- All patients that must be hospitalized should be PCR-screened. Patients who test positive for COVID-19 should receive treatment separately, according to the applicable local infection control guidelines.

#### Optimizing communication

##### With patients

- Patients with hearing impairment should be provided with:
  - a badge so that they can be visually identified.
  - an Assistive Listening Kit, such as an FM device.
  - flyers that:
    - Give instructions regarding the test procedures so that they can get an overview of the whole procedure of whatever service they will receive.
    - Have information on the communication strategies that will help them to understand the clinician during procedures.
    - Inform them about what the clinic has done to maximize safety.
- Transparent dividers can be set up at head level to enable verbal communication or lip reading.
- Hospitalized patients receive a “hearing kit” with assistive FM hearing devices.

##### With clinicians and employees of the clinic

- Regular updates and recommendations should be provided.
- Flyers describing communication strategies that can be used when wearing masks should be provided.

#### Personal protective equipment (PPE)

- Hand sanitizer should be available at multiple places, especially at the entrance and between testing rooms.
- Clinicians should wear, at minimum, a N95 respirator (or equivalent) when providing treatment; patients should wear, at minimum, a fresh cotton mask with at least 3 layers or a surgical mask. Regulations vary according to local rules, but the safest masks should be used whenever possible.
- Clinicians should wear gloves and an extra gown when treating patients with an underlying disease or who have a history of pharmacological intervention or other factors that directly influence the integrity of the immune system.
- A transparent face shield can be used (without a mask) only if there is also a transparent divider between the clinician and patient. However, a transparent face shield should never replace a mask in the absence of the transparent divider.
- Clinicians should thoroughly clean the clothes and shoes they wore in the clinic.

#### Room ventilation and room disinfection

- Rooms should be well ventilated with the direction of airflow going out of the room.
- A cassette type of air conditioner mounted on the ceiling is recommended.
- The maximum duration in the sound booth for each patient is 15 min.
- Use only the instruments in the room that you need, remove all unnecessary equipment and furniture from the room.
- The sound-treated booth/room should only be used for threshold estimation. All other procedures can be done in a well-ventilated room. (For example, while testing a child using conditioned play audiometry, training the child the conditioned response can be performed in a well-ventilated room and threshold estimation can be done in the sound-treated room).
- Rooms should be sufficiently ventilated after every 15 min of patient use.
- After each patient, rooms and equipment can be treated via appropriate UV light or gas fumigation (ethylene oxide) for at least 10 min
- Sound booth fumigation can be done using a UV lamp for 25‒30 min. As this can harm some of the components of your equipment, it is advisable to turn off the equipment during UV sterilization.
- All equipment that comes into direct contact with patients should be sterilized using alcohol wipes (70% alcohol) and a UV lamp.

### Modifications to audiological procedures

#### Overall modifications

- Do only procedures that are absolutely necessary (see Sect. “Procedures: In person or remote? Urgent or postponable?”).
- Use telerehabilitation/telehealth when possible.
- 15 min should be allotted between patients to allow the room and equipment to be ventilated/sterilized.
- Stagger appointment start times so that only 1 patient and 1 clinician are present the same time. The next patient may enter only after the testing is done and appropriate sanitation procedures are performed.
- Disinfect hands before using any equipment. This should be done in accordance with the guidance from the Centers of Disease Control and Prevention [3].

##### PTA—pure tone audiometry

- Give instruction via headphones/insert earphones or via lip reading across a screen.
- It is helpful to pre-record audio–video instructions with captions and with demonstrations of the procedure. This can be played to the patient outside the audio booth or sent to their mobile phone so that they can watch while waiting.
- Disinfect the test booth after each patient (see Sect. “Room ventilation and room disinfection”).
- Use signs outside the room that indicate if the sound booth has been cleaned yet after the procedure for the next patient to be tested.
- Depending on the age of the child, get help from parent/co-clinician to ensure they are wearing their mask properly.

##### Tympanometry

- Before testing, the patient should be given printed instructions or pre-recorded video–audio demonstration with captions.
- Use video otoscope to assess the patient’s external ear canal.
- In cases of tympanic membrane perforation, tympanometry should be avoided.

##### OAE & ABR

- Clinicians must wear appropriate PPE (gloves, mask, face shield).
- A single use tissue sheet should be placed over the couch/bed and pillow.
- Disposable electrodes should be used.

##### CI programming

- For first fitting/activation of the CI processor, the patient should be sent instructions and guidelines before the appointment
- Patients should be sent a pre-recorded demonstration with video-audio instructions with captions to explain the device components and give guidance for troubleshooting and care and maintenance. When advanced troubleshooting is necessary, the patient can leave the device at the drop off / pick-up desk at the clinic / hospital.
- A face shield with a transparent protection wall should be used to enable lip reading.

## Discussion

Vaccinations are now available, but the precautions and triage detailed herein should be the same whether a person has been vaccinated or has had COVID-19.

Because hearing is important, audiological services are important. For these services to continue under COVID-19, safety measures must be implemented and maintained, and treatment and communication strategies must be adapted to offset communication difficulties due to PPE and social distancing and to bolster patient confidence. In short, it is vital that (1) staff feel safe, (2) patients either feel the clinic is safe enough to visit or that remote treatment may be an option, and (3) clinics and patients have a broad agreement on the urgency of any needed service.

The recommendations provided in this article are based on the experience from renowned audiologists and can be used as a reference for individual clinics to set up their own operating procedures, with the consideration of the local pandemic phase.

Regarding personal safety, the importance of keeping clinicians healthy cannot be overestimated. In addition to the safety measures detailed herein and in other articles [e.g., 4,6], the judicious use of telehealth can reduce the number of people physically present in the clinic. Studies on the use of telehealth in both non-audiological patients [7] and on audiologists [8] have found that substituting telehealth for a face-to-face visit has been positively received and provides a high level of care. Telehealth is, of course, not new in audiology [e.g., 9,10] but COVID-19-related restrictions could lead to its more widespread use (and acceptance).

For cases in which telehealth is not feasible or advisable and the procedure should not be postponed, a patient visit is necessary. For this, patients must be confident that clinic visits are not dangerous if they follow the safety measures. Indeed, fear of contagion has been reported as a common reason for patients to cancel or fail to schedule clinical visits [7, 11]. It is, thus, important for clinics to not only be safe, but to be publicly perceived as safe. To this end, we believe that it is important to communicate that the safety precautions the clinic has taken. This can be done with information appealing posted inside the clinic and with information provided to patients before their visit.

Adopting effective strategies to communicate with people with hearing loss is always important but COVID-19 has made it even more important because facemasks muffle other people’s speech and make lip reading impossible [12, 13]. Several solutions have been proposed to this end, including transparent masks and portable personal amplifiers [12–14]. We advocate using Assistive Listening Kits, short videos, and flyers to augment communication, although any methods that enhance safe and effective communication with the patient could be used. Communication is vital for in health care, so it is imperative that clinics prioritize it.

In conclusion, audiological services are extremely important. Necessary services can still be provided if careful safety measures are adopted and rigorously adhered to. The pandemic has made communication more challenging but there are steps that clinics can take to meet this challenge.
